# How cystic fibrosis modulator therapy could transform Pseudomonas aeruginosa epidemiology

**DOI:** 10.1099/mic.0.001790

**Published:** 2026-10-13

**Authors:** James Gurney, Morgana Letizia, Sheyda Azimi, Stephen P. Diggle

**Affiliations:** 1 School of Biology, Georgia State University, Atlanta, Georgia, USA; 2 Center for Microbial Dynamics & Infection, School of Biological Sciences, Georgia Institute of Technology, Atlanta, Georgia, USA

**Keywords:** CFTR modulators, chronic infection, cystic fibrosis, microbial adaptation, pathogen evolution, *Pseudomonas aeruginosa*

## Abstract

The advent of cystic fibrosis transmembrane conductance regulator modulators has revolutionized cystic fibrosis (CF) care by restoring epithelial ion transport and improving airway physiology in people with CF. These therapies reduce infection burden, especially from *Pseudomonas aeruginosa*, through enhanced mucociliary clearance and immune responses. Yet, *P. aeruginosa* often persists, raising concerns about microbial adaptation. Emerging evidence suggests that modulator-altered airways impose novel selective pressures, favouring mutations in genes linked to biofilm formation, iron metabolism and immune evasion. This may drive the emergence of strains adapted to a ‘hybrid’ airway; neither fully CF-like nor fully healthy. Such adaptations could potentially increase transmissibility or virulence, with implications beyond the CF population. Understanding how pathogens evolve under modulator pressure is essential in this new treatment era. Continued surveillance of microbial dynamics will be key to anticipating shifts in infection risk and informing future clinical and public health strategies.

## Introduction

Cystic fibrosis (CF) is an autosomal recessive genetic disorder that affects multiple organs, especially the lungs and gastrointestinal tract [1]. The disease is driven by mutations in the cystic fibrosis transmembrane conductance regulator (*CFTR*) gene, with over 2,000 pathogenic variants identified to date. *CFTR* encodes a protein responsible for regulating the movement of chloride and bicarbonate ions across epithelial cell membranes. In people with CF (pwCF), this protein is often absent, misfolded or dysfunctional, depending on the specific mutation. One consistent result is that chloride and water transport is disrupted across multiple organs, resulting in the production of thick, dehydrated mucus that clogs airways and ducts. In the lungs, this mucus results in reduced airway surface liquid, impaired mucociliary clearance and bacterial entrapment, leading to chronic lung infections, persistent inflammation, airway damage (bronchiectasis) and, over time, progressive respiratory failure [1] (Fig. 1).

**Fig. 1.**
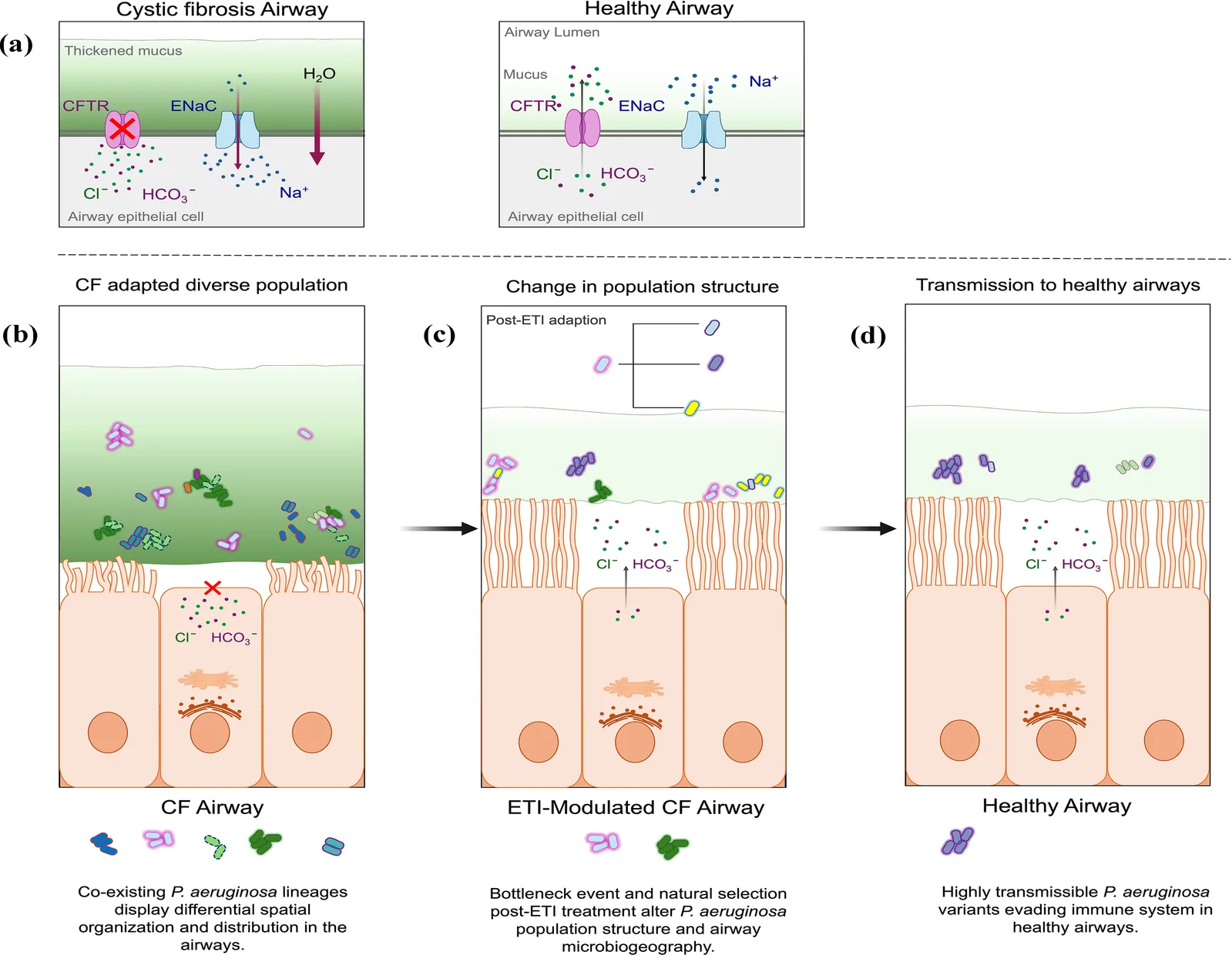
*Pseudomonas aeruginosa* population structure and evolutionary trajectories in the modulator era. (**a**) Pathophysiology of CF airway. F508del is the most common CFTR mutation that causes CFTR misfolding and prevents trafficking to the plasma membrane. Mutations in CFTR disrupt Cl^−^ and HCO_3_^−^ transport, resulting in dehydration and buildup of thick, sticky mucus compared to healthy airways. (**b**) The emergence and accumulation of pathoadaptive mutations is a hallmark of chronic CF airway infections. The co-existence of diverse *P. aeruginosa* lineages and intra-population allelic polymorphism modulates spatial organization and niche distribution in CF airways. (**c**) The modulator-induced bottleneck. Modulator treatments improve CFTR processing and trafficking and increase the amount of functional protein at the membrane, resulting in significant changes in airway physicochemical properties. Although this bottleneck decreases *P. aeruginosa* burden, it also alters the population structure where genetic variants with the ability to withstand the new environment persist in airways. (**d**) Future adaptive trajectories. Hypothetical outcome in which *P. aeruginosa* adaptation to modulator-treated airways leads to emergence and accumulation of variants with increased fitness to transmit to and colonize a healthy, non-CF host.

CF care is being transformed by the advent of CFTR modulators, such as the triple therapy elexacaftor/tezacaftor/ivacaftor (ETI), commercially known as Trikafta in the USA or Kaftrio in Europe [2, 3] or the newly approved triple-combination modulator vanzacaftor/tezacaftor/deutivacaftor, known as Alyftrek. Collectively, these drugs are known as highly effective modulator therapy (HEMT) [4]. HEMT targets the root cause of CF by partially correcting the dysfunctional CFTR protein, thereby improving chloride and bicarbonate transport across epithelial cells [5]. Clinically, this translates into dramatic improvements in lung function, mucus hydration, immune regulation, reduced inflammation and improved airway clearance [2–4, 6–9]. These improvements profoundly enhance patient quality of life, significantly reducing symptom burden, treatment complexity and overall healthcare demands. Over 75,000 pwCF worldwide are currently receiving a CFTR modulator, representing around two-thirds of those eligible. Eligibility and access are, however, distinct constraints: ∼10–15% of pwCF are ineligible on genotype, age or other grounds [10], while a further substantial proportion of eligible individuals remain untreated owing to reimbursement and geographic disparities. A globally significant reservoir of unmodulated, chronically infected airways, therefore, persists alongside the modulator-treated population.

For many pwCF, the lung environment is now trending toward a more physiologically healthy state. However, despite these advances, early studies show that pathogens, including *Pseudomonas aeruginosa* [11, 12], often persist in the lungs of pwCF on modulators [13–17]. This persistence raises an intriguing and potentially important question: could the modulator-treated CF lung, with its improved mucus clearance, immune response and epithelial function, exert new selective pressures that drive *P. aeruginosa* to evolve in unexpected ways? Specifically, given the well-documented within-host adaptation trajectories of *P. aeruginosa* populations, might this new environment favour traits that not only promote survival in modulator-altered lungs but also increase the pathogen’s ability to infect individuals without CF, particularly patients with non-CF bronchiectasis? In this perspective, we discuss how the lung’s microbial ecology is shifting in response to correctors and whether these changes could inadvertently ‘train’ *P. aeruginosa* to become a more versatile and potentially more widespread pathogen.

### Shifting microbial dynamics with modulator therapy

One immediate effect of modulator-improved clearance is a change in the lung microbiology. PwCF traditionally had a predictable progression in lung infections, initially infected with either *Haemophilus influenzae* or *Staphylococcus aureus* and typically progressing into being chronically infected with *P. aeruginosa*. After HEMT, overall bacterial burden in the airways is declining, as evidenced by reductions in the frequency of pathogenic detection, decreased reliance on antibiotic therapy and diminished need for airway clearance interventions, such as chest physiotherapy [15, 16, 18]. Modulator therapy impacts the broader microbial community, reducing overall pathogen diversity and potentially altering interspecies interactions. Early initiation of modulators, especially in paediatric and adolescent populations, may entirely prevent the establishment of chronic infections by maintaining functional airway defences [19–21]. These developments have led some clinicians to envision a future in which chronic colonization with pathogens, such as *P. aeruginosa* may become rare or even absent in the CF population. However, pathogens have been demonstrated to persist in study participants initiating Trikafta in adolescence, which means there may be at least a generation for whom Trikafta does not clear all bacteria [16].

Following treatment with the first available modulator, ivacaftor, *P. aeruginosa* infections persisted in pwCF [22, 23], and pathogen density rebounded after ∼1–2 years in chronically infected adults post-HEMT [22]. According to recent findings from the 27-centre prospective PROMISE-Micro study, the average density of *P. aeruginosa* in sputum from chronically infected individuals decreases ∼tenfold within the first month of Trikafta therapy. However, on average, no further significant decline was observed over the subsequent 3.5 years. Only ~10% of participants who were culture-positive at baseline prior to Trikafta treatment became repeatedly culture-negative after 3.5 years. Notably, *P. aeruginosa* density patterns varied widely among participants. In general, a predictable relationship appears to exist between baseline *P. aeruginosa* density and the percentage of participants becoming repeatedly culture-negative, with chronic infections being the most difficult to eradicate [15]. Similar trends have been reported in multiple studies [9, 17, 24, 25]. In one recent study, *P. aeruginosa* persisted after Trikafta therapy in 18 of 19 chronically colonized adults [13]. Notably, the same clonal strains present before treatment were found post-treatment, suggesting survival under therapeutic pressure instead of replacement [13, 26]. Rather than clearing the lung of pathogens, CFTR modulators appear to shift the ecological landscape, rendering it less permissive to some bacterial species and thereby imposing different selective pressures than before. This altered environment, if it more closely resembles that of individuals without CF, may drive adaptive evolution in *P. aeruginosa*, as they adjust to new host defences and airway conditions (Fig. 1).

### *P. aeruginosa* adaptation to the CF lung in the pre-modulator era

Before the introduction of CFTR modulators, *P. aeruginosa* already exhibited a remarkable ability to adapt to the distinctive pathophysiological environment of the CF lung [27–30]. *P. aeruginosa* undergoes a well-documented phenotypic shift from an acute, planktonic lifestyle to a chronic, biofilm-based mode of growth [31]. This transition includes down-regulation or loss of energetically costly acute-infection-phase traits, such as flagellar motility and type III secretion system components and up-regulation of traits promoting long-term survival, such as alginate biosynthesis [28]. Alginate contributes to the mucoid biofilm phenotype that physically shields bacterial cells from both host immune responses and antibiotic penetration [32]. Pre-HEMT genomic studies of longitudinally sampled clonal lineages revealed repeated accumulation of mutations in genes associated with LPS biosynthesis (e.g. O-antigen truncation), nutrient acquisition, efflux pump regulation and quorum sensing [33]. These adaptations collectively enhance survival in the hypoxic, inflamed and antibiotic-laden environment of the CF airway.

The resulting phenotypes represent evolutionary adaptation and trade-offs, resulting in a highly specialized pathogen optimized for persistence within the CF lung [33], but somewhat attenuated in the capacity for transmission or even acute infection [27]. It should be noted that some *P. aeruginosa* strains are classified as epidemic and transmissible in CF populations due to their ability to spread from patient to patient, often within clinical settings. These strains, which include the Liverpool Epidemic Strain (LES) and others, have been associated with increased antibiotic resistance, enhanced virulence and worse clinical outcomes and have been documented to colonize multiple individuals across regions and countries [33, 34]. A proportion of pwCF, therefore, acquired their *P. aeruginosa* strain directly from another person with CF. Epidemic strains are informative here because they already occupy the phenotypic space we hypothesize the post-HEMT lung might select for. LES isolates combine chronic-infection traits with retained or elevated expression of virulence determinants, including early overproduction of quorum sensing-regulated exoproducts [33, 34], can super-infect and displace resident populations in already colonized airways and have been transmitted to non-CF relatives of pwCF [35]. A question for the modulator era is whether the hybrid airway makes such phenotypes more likely to arise, through resident lineages regaining acute traits or to establish, through acquisition of strains that already possess them. The second route deserves attention, since the post-modulator bottleneck reduces standing diversity and should lower the resident population’s resistance to invasion.

### Ecological disruption in the modulator era

The physiological changes mediated by HEMT constitute a substantial shift in the selective pressures acting on persistent pathogens like *P. aeruginosa* [13, 14, 36]. For a thorough review of the impacts on the CF lung microbiome post-HEMT, we suggest Robertson *et al*. [32]. While there are potentially many differences between pre- and post-HEMT lungs, we propose three changes that reshape the lung ecological landscape and could promote patient-to-patient transmission: (i) decreased mucus viscosity, limiting biofilm-stabilizing niches and reducing aggregate formation; (ii) enhanced ciliary activity, promoting bacterial clearance; (iii) increased immune surveillance; and (iv) a marked reduction in antibiotic exposure, as exacerbation frequency and reliance on chronic suppressive therapy decline. The consequences for antimicrobial therapy are worth considering and are not necessarily unfavourable. These lung changes could result in a shift toward more planktonic, faster-growing populations and should, in principle, restore the efficacy of agents, such as aminoglycosides and β-lactams, whose activity is growth-rate dependent. Reduced antibiotic exposure may also relax selection for resistance determinants and for hypermutability. Collectively, these factors create a more dynamic and less permissive environment, potentially selecting for *P. aeruginosa* variants with distinct fitness advantages under post-modulator conditions (Fig. 1).

Evidence is beginning to support this evolutionary hypothesis. Two recent studies have identified variants of concern in post-modulator isolates. Ledger *et al*. found that genes previously implicated in classical CF adaptation, including those regulating alginate biosynthesis (*mucA*, *algG*), biofilm architecture (*pslA*, *pelA*, *cdrA*), motility (*flgK*) and iron acquisition (*pvdA*, *pvdL*), were under selection [14]. In a similar analysis, three iron-related genes were found to be under selection (*feoB*, *pvdQ* and PA2062) [13]. The authors identified a population bottleneck immediately following modulator exposure, likely driven by enhanced host defences and subsequent expansion of a reduced bacterial subpopulation. It is important to note that none of these loci are novel to *P. aeruginosa* evolution within human hosts and have been previously described in pwCF and non-CF individuals [33]. What may be new is the direction and timing of selection rather than the targets, and although these genes are already known to be under selection in pwCF, surviving lineages in post-HEMT lungs could carry new mutations, and the changes detected to date appear to track the altered airway environment closely. For example, increased iron levels have been observed in the upper respiratory tract of pwCF undergoing Trikafta therapy, serving as a biomarker positively associated with improved pulmonary function [37].

The recurrence of mutations across multiple patients suggests convergent evolution in response to shared selective forces in the modulator-treated lung. In both studies, changes in iron acquisition seem to occur, suggestive of an improved nutritional immunity selecting for *P. aeruginosa* with alternative iron scavenging strategies. Notably, additional mutations affected LPS pathways, suggesting remodelling of surface structures that may influence immune recognition. Mutations in *flgK* have previously been shown to provide resistance to phagocytosis [38]. Despite these adaptations, hallmark traits of chronic infection (e.g. mucoidy, antibiotic resistance) often persist, highlighting the pathogen’s capacity to retain core persistence mechanisms while evolving under new selective pressures. Moreover, bulk transcriptomic analyses comparing *P. aeruginosa* gene expression in human CF sputum samples collected before and after ETI initiation suggest that, so far, the CF airway continues to support a similar bacterial transcriptional state despite modulator therapy [39].

### Could the modulated CF lung promote cross-host adaptation in *P. aeruginosa*?

An important and as-yet unresolved question is whether *P. aeruginosa* evolution in modulator-treated lungs could produce strains with increased capacity to infect non-CF individuals, such as those with non-CF bronchiectasis (Fig. 1). While the post-modulator lung is not fully normalized, it is more permissive to mucociliary clearance and immune function than the classic CF airway [3]. This ‘hybrid’ environment could act as a selective filter, favouring strains that maintain or regain certain virulence-associated traits, such as motility, toxin production or stress resistance, without completely shedding chronic adaptations [14, 33]. Historically, CF-adapted strains exhibit reduced virulence in non-CF hosts due to mutations in regulators, such as *lasR* and *retS*, which down-regulate acute virulence phenotypes. However, if modulators reverse some of these pressures, strains with intact or compensated regulatory pathways may gain fitness advantages. This could lead to the emergence of mixed-phenotype populations, some of which may resemble environmental or opportunistic *P. aeruginosa* strains capable of causing disease in immunocompromised or hospitalized individuals.

Although this scenario remains highly speculative and there is currently no evidence of increased transmission from pwCF on modulators to non-CF individuals, it is worth monitoring the evolutionary trajectory of persistent *P. aeruginosa* strains in this new airway environment. The restored airway conditions may favour strains with altered phenotypes, and rare transmission events, while unlikely, could have future implications if accompanied by enhanced virulence or resistance. However, we acknowledge that HEMT has been shown to reduce bacterial burden. Furthermore, sputum production is often greatly reduced in most modulator-treated individuals, which again is likely to diminish transmission risk.

## Conclusion

The introduction of CFTR modulators has marked a key shift in the management of CF, allowing therapy to move from a reactive position of mitigating symptoms to direct correction of the underlying pathophysiology [1, 3]. These therapies have undoubtedly shifted the host–pathogen balance in favour of pwCF. However, this progress brings new considerations: as the lung environment is altered, it imposes novel selective pressures on persistent microbial populations, most notably *P. aeruginosa*, which may respond through adaptive evolution.

Emerging evidence indicates that *P. aeruginosa* can persist in modulator-treated airways by acquiring new mutations, potentially altering its phenotype in response to this changing niche [13]. While current data suggest that most patients experience reduced bacterial loads and improved airway function, the evolutionary consequences of these shifts remain uncertain. A critical question is whether these adaptations might inadvertently select traits that enhance the pathogen’s capacity to infect hosts outside the CF population. Conceptually, the modulator-treated lung could act as a ‘training ground’, selecting for strains with increased fitness in environments becoming distinct from CF.

We may be entering a new chapter in CF microbiology, one in which the pathogens are evolving in response to therapies that do not directly target them. Vigilant monitoring of microbial dynamics in this therapeutic era is warranted. This includes genomic surveillance of persistent isolates, characterization of emergent phenotypes and assessment of transmission and virulence potential. Although most evidence thus far points to a reduction in pathogen prevalence, particularly with earlier intervention, the evolutionary plasticity of *P. aeruginosa* underscores the need for continued observation. Proactive clinical strategies, including routine genomic surveillance, early microbiological monitoring and personalized antimicrobial regimens, could mitigate these evolutionary risks and optimize patient outcomes in the era of CFTR modulator therapy. Ultimately, the hope is that with earlier and widespread modulator use, and with future more effective modulator drugs, chronic *P. aeruginosa* infections in pwCF will become a rarity, firmly closing the door on this evolutionary drama altogether.

## Abbreviations

CF: cystic fibrosis
CFTR: cystic fibrosis transmembrane conductance regulator
ETI: elexacaftor/tezacaftor/ivacaftor
HEMT: highly effective modulator therapy
LES: Liverpool Epidemic Strain
pwCF: people with CF

## References

[1] Elborn JS (2016). Cystic fibrosis. The Lancet.

[2] Heijerman HGM, McKone EF, Downey DG, Van Braeckel E, Rowe SM (2019). Efficacy and safety of the elexacaftor plus tezacaftor plus ivacaftor combination regimen in people with cystic fibrosis homozygous for the F508del mutation: a double-blind, randomised, phase 3 trial. Lancet.

[3] Middleton PG, Mall MA, Dřevínek P, Lands LC, McKone EF (2019). Elexacaftor-tezacaftor-ivacaftor for cystic fibrosis with a single Phe508del allele. N Engl J Med.

[4] Caverly LJ, Riquelme SA, Hisert KB (2022). The impact of highly effective modulator therapy on cystic fibrosis microbiology and inflammation. Clin Chest Med.

[5] Lopes-Pacheco M (2019). CFTR modulators: the changing face of cystic fibrosis in the era of precision medicine. Front Pharmacol.

[6] Ridley K, Condren M (2020). Elexacaftor-tezacaftor-ivacaftor: the first triple-combination cystic fibrosis transmembrane conductance regulator modulating therapy. J Pediatr Pharmacol Ther.

[7] Nichols DP, Paynter AC, Heltshe SL, Donaldson SH, Frederick CA (2022). Clinical effectiveness of elexacaftor/tezacaftor/ivacaftor in people with cystic fibrosis: a clinical trial. Am J Respir Crit Care Med.

[8] Bower JK, Volkova N, Ahluwalia N, Sahota G, Xuan F (2023). Real-world safety and effectiveness of elexacaftor/tezacaftor/ivacaftor in people with cystic fibrosis: Interim results of a long-term registry-based study. J Cyst Fibros.

[9] Sheikh S, Britt RD, Ryan-Wenger NA, Khan AQ, Lewis BW (2023). Impact of elexacaftor-tezacaftor-ivacaftor on bacterial colonization and inflammatory responses in cystic fibrosis. Pediatr Pulmonol.

[10] Desai M, Hine C, Whitehouse JL, Brownlee K, Charman SC (2022). Who are the 10%? - Non eligibility of cystic fibrosis (CF) patients for highly effective modulator therapies. Respir Med.

[11] Diggle SP, Whiteley M (2020). Microbe Profile: *Pseudomonas aeruginosa*: opportunistic pathogen and lab rat. *Microbiology (Reading*).

[12] Letizia M, Diggle SP, Whiteley M (2025). *Pseudomonas aeruginosa*: ecology, evolution, pathogenesis and antimicrobial susceptibility. Nat Rev Microbiol.

[13] Armbruster CR, Hilliam YK, Zemke AC, Atteih S, Marshall CW (2024). Persistence and evolution of *Pseudomonas aeruginosa* following initiation of highly effective modulator therapy in cystic fibrosis. mBio.

[14] Ledger EL, Smith DJ, Teh JJ, Wood ME, Whibley PE (2024). Impact of CFTR modulation on *Pseudomonas aeruginosa* infection in people with cystic fibrosis. J Infect Dis.

[15] Morgan SJ, Coulter E, Betts HL, Solomon GM, Clancy JP (2024). Elexacaftor/tezacaftor/ivacaftor’s effects on cystic fibrosis infections are maintained, but not increased, after 3.5 years of treatment. J Clin Invest.

[16] Nichols DP, Morgan SJ, Skalland M, Vo AT, Van Dalfsen JM (2023). Pharmacologic improvement of CFTR function rapidly decreases sputum pathogen density, but lung infections generally persist. J Clin Invest.

[17] Schnell A, Hober H, Kaiser N, Ruppel R, Geppert A (2023). Elexacaftor - Tezacaftor - Ivacaftor treatment improves systemic infection parameters and *Pseudomonas aeruginosa* colonization rate in patients with cystic fibrosis a monocentric observational study. Heliyon.

[18] Mianowski L, Doléans-Jordheim A, Barraud L, Rabilloud M, Richard M (2024). One year of ETI reduces lung bacterial colonisation in adults with cystic fibrosis. Sci Rep.

[19] Toraih EA, Malik HA, Islam RK, Pirzadah HA, Abdelmaksoud A (2025). Clinical outcomes in patients with cystic fibrosis receiving CFTR modulators: a comparison of childhood versus adolescent initiation. Children (Basel).

[20] Duehlmeyer S, Elson EC, Oermann CM (2024). Effect of Elexacaftor/Tezacaftor/Ivacaftor on *Pseudomonas aeruginosa* acquisition and chronic infection at a single pediatric cystic fibrosis care center. J Pediatr Pharmacol Ther.

[21] Steinberg R, Moeller A, Gisler A, Mostacci N, Hilty M (2025). Longitudinal effects of elexacaftor/tezacaftor/ivacaftor on the oropharyngeal metagenome in adolescents with cystic fibrosis. J Cyst Fibros.

[22] Hisert KB, Heltshe SL, Pope C, Jorth P, Wu X (2017). Restoring cystic fibrosis transmembrane conductance regulator function reduces airway bacteria and inflammation in people with cystic fibrosis and chronic lung infections. Am J Respir Crit Care Med.

[23] Heltshe SL, Mayer-Hamblett N, Burns JL, Khan U, Baines A (2015). *Pseudomonas aeruginosa* in cystic fibrosis patients with G551D-CFTR treated with ivacaftor. Clin Infect Dis.

[24] Beck MR, Hornick DB, Pena TA, Singh SB, Wright BA (2023). Impact of elexacaftor/tezacaftor/ivacaftor on bacterial cultures from people with cystic fibrosis. Pediatr Pulmonol.

[25] Durfey SL, Kapnadak SG, Pena T, Willmering MM, Godwin JD (2025). Pseudomonas infections persisting after CFTR modulators are widespread throughout the lungs and drive lung inflammation. Cell Host Microbe.

[26] Ledger EL, Smith DJ, Teh JJ, Wood ME, Whibley PE (2024). Impact of CFTR modulation on pseudomonas aeruginosa infection in people with cystic fibrosis. J Infect Dis.

[27] Smith EE, Buckley DG, Wu Z, Saenphimmachak C, Hoffman LR (2006). Genetic adaptation by *Pseudomonas aeruginosa* to the airways of cystic fibrosis patients. Proc Natl Acad Sci U S A.

[28] Folkesson A, Jelsbak L, Yang L, Johansen HK, Ciofu O (2012). Adaptation of *Pseudomonas aeruginosa* to the cystic fibrosis airway: an evolutionary perspective. Nat Rev Microbiol.

[29] Rossi E, La Rosa R, Bartell JA, Marvig RL, Haagensen JAJ (2021). *Pseudomonas aeruginosa* adaptation and evolution in patients with cystic fibrosis. Nat Rev Microbiol.

[30] Vanderwoude J, Azimi S, Read TD, Diggle SP (2024). The role of hypermutation and collateral sensitivity in antimicrobial resistance diversity of *Pseudomonas aeruginosa* populations in cystic fibrosis lung infection. mBio.

[31] Bjarnsholt T, Alhede M, Alhede M, Eickhardt-Sørensen SR, Moser C (2013). The in vivo biofilm. Trends Microbiol.

[32] Robertson JK, Goldberg JB (2025). The impact of cystic fibrosis transmembrane conductance regulator (CFTR) modulators on the pulmonary microbiota. *Microbiology (Reading*).

[33] Weimann A, Dinan AM, Ruis C, Bernut A, Pont S (2024). Evolution and host-specific adaptation of *Pseudomonas aeruginosa*. Science.

[34] Salunkhe P, Smart CHM, Morgan JAW, Panagea S, Walshaw MJ (2005). A cystic fibrosis epidemic strain of *Pseudomonas aeruginosa* displays enhanced virulence and antimicrobial resistance. J Bacteriol.

[35] McCallum SJ, Gallagher MJ, Corkill JE, Hart CA, Ledson MJ (2002). Spread of an epidemic *Pseudomonas aeruginosa* strain from a patient with cystic fibrosis (CF) to non-CF relatives. Thorax.

[36] Sosinski LM, H CM, Neugebauer KA, Ghuneim L-AJ, Guzior DV (2022). A restructuring of microbiome niche space is associated with Elexacaftor-Tezacaftor-Ivacaftor therapy in the cystic fibrosis lung. J Cyst Fibros.

[37] Atteih SE, Armbruster CR, Hilliam Y, Rapsinski GJ, Bhusal JK (2024). Effects of highly effective modulator therapy on the dynamics of the respiratory mucosal environment and inflammatory response in cystic fibrosis. Pediatr Pulmonol.

[38] Amiel E, Lovewell RR, O’Toole GA, Hogan DA, Berwin B (2010). *Pseudomonas aeruginosa* evasion of phagocytosis is mediated by loss of swimming motility and is independent of flagellum expression. Infect Immun.

[39] Letizia M, Whiteley L, Diggle F, McKone E, Singh P (2026). The impact of modulator therapy on the *Pseudomonas aeruginosa* CF lung transcriptome. PNAS (in press).

